# SeXX Matters in Infectious Disease Pathogenesis

**DOI:** 10.1371/journal.ppat.1005374

**Published:** 2016-02-18

**Authors:** Landon G. vom Steeg, Sabra L. Klein

**Affiliations:** The W. Harry Feinstone Department of Molecular Microbiology and Immunology, The Bloomberg School of Public Health, Johns Hopkins University, Baltimore, Maryland, United States of America; Duke University Medical Center, UNITED STATES

## Sex Versus Gender Differences

We often use the terms “sex” and “gender” interchangeably in infectious diseases research, which is incorrect because these terms refer to different aspects of biology and behavior. The term “sex” refers to biological characteristics that define males and females, including the basic organization of chromosomes, reproductive organs, and circulating sex steroid hormone concentrations. Gender refers to the roles, behaviors, and activities that are defined by social or cultural norms, including gender norms associated with education, occupation, and health-seeking behaviors [1].

If we consider infectious diseases in the context of sex and gender, then we could hypothesize that sex results in physiological differences (e.g., hormonal regulation of immune responses) that contribute to male–female differences in the control and clearance of a pathogen as well as anatomical differences that may affect exposure and transmission of a pathogen. Gender is likely associated with behaviors that influence differential exposure to pathogens. Gender also contributes to the norms that affect access to health care and health-seeking behaviors, which could influence male–female differences in the duration and severity of infection in some countries [1]. By and large, both the intensity (i.e., pathogen load within an individual) and prevalence (i.e., number of infected individuals within a population) of infections are often higher for males than females, illustrating that both sex and gender play roles in male–female differences in infectious disease pathogenesis.

## Evidence of Male–Female Differences in Infectious Diseases

**Fig 1:**
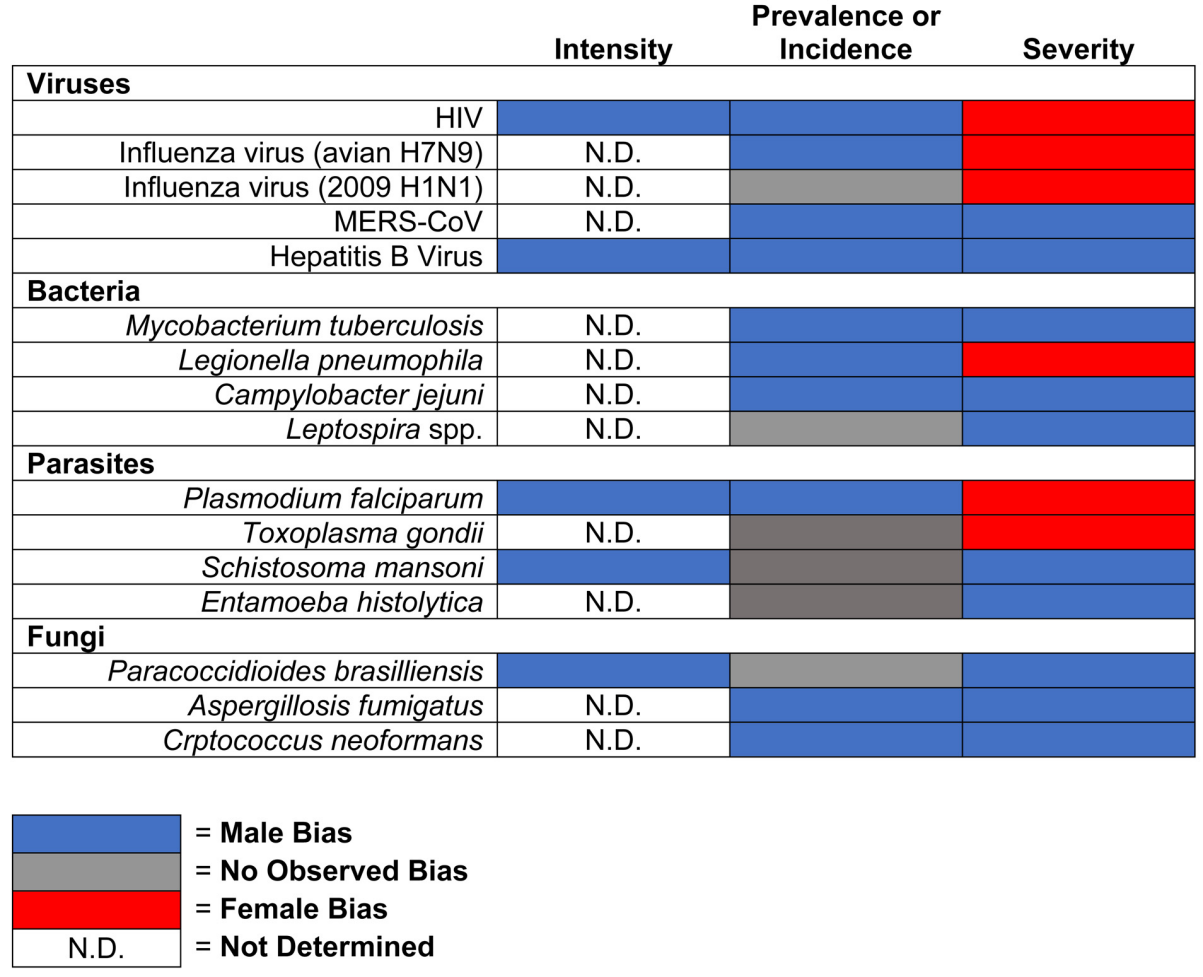
Sex differences in the intensity (i.e., pathogen load), prevalence (i.e., proportion of population with disease), incidence (i.e., new cases of disease), and severity (i.e., hospitalization or progression of disease state) of disease following microbial infections in humans.

The sexes differ in the intensity, prevalence, and pathogenesis of infections caused by viruses, bacteria, parasites, and fungi. Males and females of species ranging from humans to horses and rodents differ in their responses to and the outcome of diverse pathogenic infections (Fig 1) [2,3]. For each of these infectious diseases, there are numerous and diverse ways in which sex and gender can impact differential susceptibility between males and females. For example, human studies reveal that women have over 40% less human immunodeficiency virus (HIV) RNA in circulation than men. Despite having less circulating HIV RNA than men, women who are matched with men on their HIV RNA loads have a 1.6-fold higher risk of developing AIDS [4]. Although exposure to influenza A viruses is often higher in men, fatality following exposure to pathogenic influenza A viruses is reportedly higher in women [1]. In contrast, the prevalence of serum hepatitis B virus (HBV) surface antigen, HBV DNA titers, and development of hepatocellular carcinoma is higher in men than women [5]. In most countries, tuberculosis notification is two times higher for men than women [6]. In tropical and sub-tropical countries (as well as in travelers to those countries), 80% of patients with amebic liver abscess, caused by the protozoan parasite *Entomoeba histolytica*, are men [7]. Among immunocompromised patients, clinical cryptococcosis is ten times higher for men than women [8]. As a general rule, males are more susceptible to infection with diverse pathogens than females (Fig 1), but the underlying causes for greater susceptibility in males are diverse.

## Anatomical Differences between the Sexes and Infectious Disease Susceptibility

The anatomy of the female genital tract can make transmission of certain infections to females more efficient than to males. The surface area of the cervicovaginal mucosa in females is larger than that of the penis and foreskin in males [4]. Damage to the mucosa epithelium during intercourse is greater for females than males [4]. Additional characteristics of the genital mucosa epithelium, including epithelium thickness, the frequency of Langerhans cells (i.e., dendritic cells [DCs] that present antigens in the skin), and the presence of lactobacilli differ between the sexes and are altered by sex steroid hormones [4]. Sex differences in the mucosal epithelium outside of the reproductive tract, e.g., in the gut and lungs, have not been systematically evaluated to explain sex differences in the intensity or prevalence of infections at these mucosal sites.

## Sex Differences in Immune Function

Sex differences in the pathogenesis of infectious diseases may reflect differences in the immune responses during infection. Males and females differ in their innate immune responses, suggesting that some sex differences are germ line-encoded. Innate detection of nucleic acids by pattern recognition receptors (PRRs) differs between the sexes [9]. There are differences between the sexes in the induction of genes associated with antiviral responses, with immune cells from females showing a 10-fold greater level of expression than cells from males [10]. Studies of both humans and rodents illustrate that the number and activity of innate immune cells, including macrophages and DCs as well as inflammatory immune responses, are higher in females than males [11–13].

Generally, females exhibit greater antibody and cell-mediated immune responses to antigenic stimulation, vaccination, and infection than do males. Both basal levels of immunoglobulin and antibody responses to pathogens and vaccines are higher in females than males [10]. Clinical studies reveal that men have lower CD3^+^ and CD4^+^ T cell counts, CD4^+^ to CD8^+^ cell ratios, and Th1 responses than women [14,15]. Females exhibit higher cytotoxic T cell activity along with upregulated expression of antiviral and proinflammatory genes, many of which have estrogen response elements in their promoters [16].

**Fig 2:**
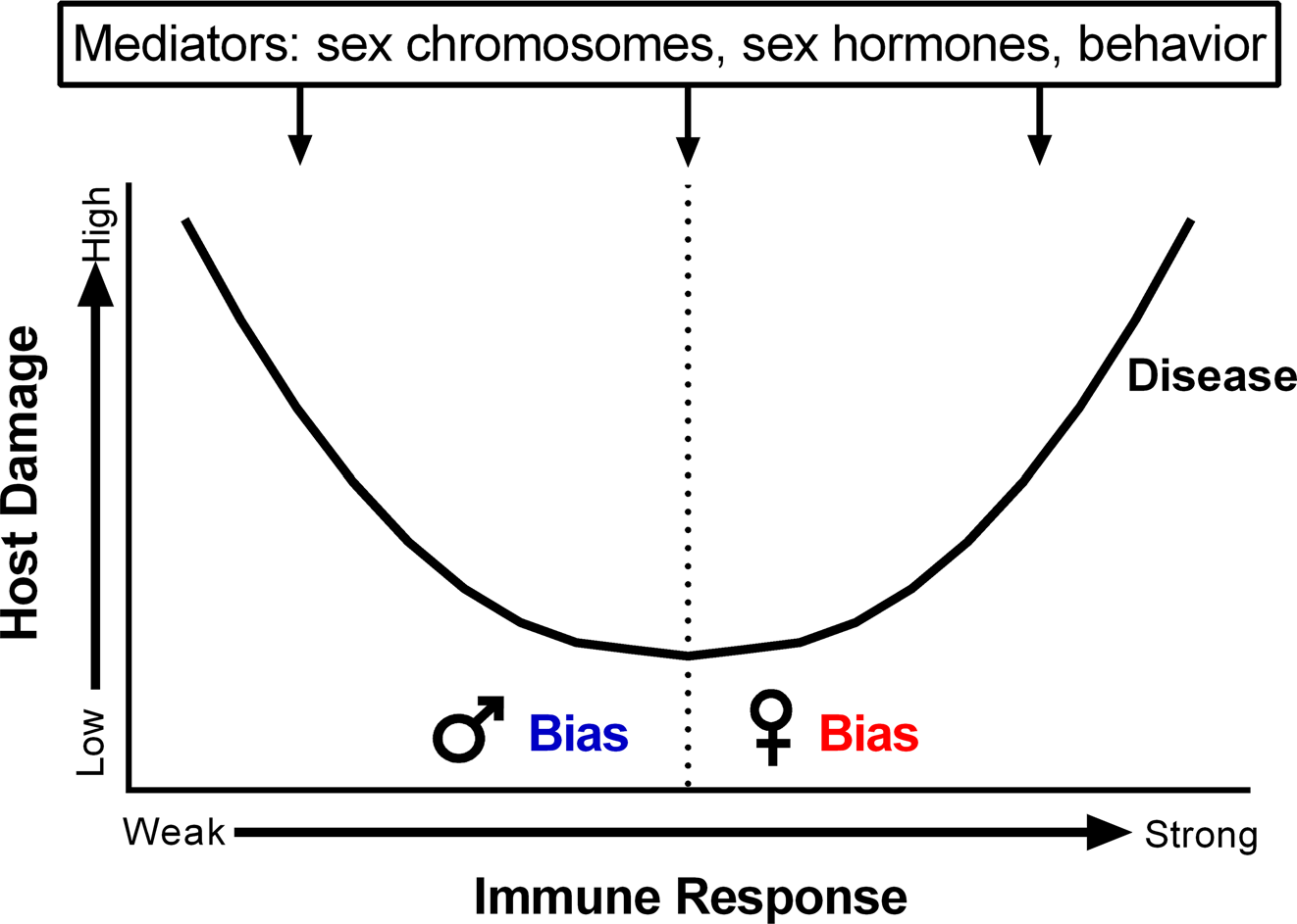
The hypothesized relationship between host damage and the host immune response following infection, as presented in the context of the damage-response framework [17]. When an immune response is “weak” (i.e., not sufficient to clear a pathogen), the damage caused by infection is high, and when an immune response is too “strong” (i.e., excessive enough to cause tissue damage), the damage caused by infection can also be high. Based on the literature presented, we hypothesize that a male bias in disease risk may be observed when weak immune responses underlie high levels of host damage and a female bias in disease risk may be observed when strong immune responses contribute to host damage. Several host factors, including sex chromosomal complement, concentrations of sex hormones, and behaviors can contribute to biases in the outcome of infection.

A future challenge will be to interpret immunological differences between the sexes in the context of infectious disease pathogenesis. In some cases, heightened antiviral, inflammatory, and cellular immune responses in females, though essential for pathogen clearance, may underlie increased development of symptoms of disease among females as compared with males following infection (Fig 1). By contrast, for other infectious diseases, an inability to properly clear or control a pathogen may contribute to increased severity of disease in males as compared with females (Fig 1). Future studies may interpret sex differences in infectious disease pathogenesis in the context of the “damage-response” framework [17], recognizing that the underlying causes of microbial pathogenesis may be mediated by the host immune response, the pathogen, or both (Fig 2).

## Mechanisms of Sex Differences in Infectious Disease Pathogenesis

### Sex steroids

Sex steroids, specifically testosterone, estrogens, and progesterone, occur in different concentrations between the sexes, with males typically having greater levels of testosterone and females often having greater levels of estrogen and progesterone at reproductive ages. Concentrations of sex steroids also differ between the sexes during perinatal development and during reproductive senescence, but not to the same extent as during the years between puberty and reproductive senescence.

Some pathogens can respond directly to host sex steroids. Sex steroids can alter the composition of commensal bacteria in the gut to cause sex-specific development of disease [18]. The genome of human papillomavirus (HPV) high-risk type 16 and 18 contains a progesterone response element (PRE). When progesterone activates the PRE, this regulates part of the HPV life cycle and transformation process, which may explain the higher frequency of malignant HPV lesions in females compared with males [19]. *Candida albicans* contains an estrogen-binding protein that has a high affinity for estradiol, which can stimulate transition of the yeast into a hyphal form that may increase fungal virulence [20].

Pathogens can directly alter concentrations of sex steroids in their host. Female mice are more susceptible to infection with *Taenia crassiceps* than males, in part because estradiol enhances parasite reproduction. In male rodents, *T*. *crassiceps* can enzymatically reduce both serum and testicular testosterone concentrations and increase estradiol concentrations to promote its own reproduction [21]. Other pathogens, including *Schistosoma mansoni*, can synthesize sex steroids and possess classical sex steroid receptors [21]. How pathogens utilize and even produce sex steroids to promote replication and transmission differentially between the sexes requires consideration.

Sex steroids can influence the functioning of host immune cells by binding to specific receptors that are expressed in most immune cells, including lymphocytes, macrophages, and DCs [22]. Sex steroids cause a majority of their cellular effects by binding to receptors located in the cytoplasm. Once bound, the hormone-receptor complex translocates to the nucleus of the cell and binds to segments of DNA that contain specific hormone response elements (HREs). The binding of sex steroids to their respective steroid receptors directly influences signaling pathways associated with the production of cytokines and chemokines [22]. Genes that encode for immunological proteins (e.g., IFNγ) can have HREs in their promoters, allowing for sex hormone receptors to act as transcriptional factors directly altering gene expression [23].

### Sex chromosome-linked genes

Some sex differences may be caused by the inherent imbalance in the expression of genes encoded on the X and Y chromosomes of a host. There is greater activation of X-linked genes in immune cells from females than males following damage [24]. Many genes on the X chromosome regulate immune function and play an important role in modulating sex differences in the development of immune-related diseases. The PRR *Tlr7* is located on the X chromosome, recognizes viruses with RNA genomes, and has higher expression levels in cells from female than males [25]. DCs isolated from women produce twice as much IFN-α in response to TLR7 ligands, including HIV-1–encoded TLR7 ligands, than do DCs from men [26]. Polymorphisms in Y chromosome genes also affect sex-dependent susceptibility to autoimmune disease [27]. The expression of X-linked genes may also be affected by X-linked microRNAs. There are a disproportionately higher number of microRNAs located on the X chromosome than on any autosomal chromosome, which is hypothesized to contribute to sex-specific development of immune-mediated diseases [28]. Interpretation of sex differences in the expression of X-linked genes is challenging because sex hormones or sex chromosome complement can still contribute to the observed differential gene expression.

## Concluding Remarks

Sex differences in response to pathogens are evolutionarily well conserved, being present across diverse host and pathogen species. The sexes provide different genetic backgrounds, anatomic niches, immunological profiles, and hormonal environments that can directly affect pathogens as well as the development of diseases following infection. Hormones, genes, and behaviors contribute significantly to sex differences in the outcome of infection (Fig 2).

In most cases, we do not know the precise mechanism mediating the dimorphism in infectious disease pathogenesis, partly because sex has not been considered a biological variable for the analysis of outcome data. The status quo is to assume that the sexes do not differ, which has hindered our understanding of the pathogenesis of infectious diseases and the underlying mechanisms. To remedy this situation, the National Institutes of Health (NIH) announced policies (NIH notice number: NOT-OD-15-102) that “require applicants to report their plans for the balance of male and female cells and animals in preclinical studies in all future applications, unless sex-specific inclusion is unwarranted, based on rigorously defined exceptions.” Some journals also have established policies that require authors to report the sex of their cells, animals, and subjects. By including sex in the analysis of outcome data, we may better understand the mechanisms governing infectious diseases and treatments for these diseases.
